# Supplementary material for: Exact Partial Information Decompositions for Gaussian Systems Based on Dependency Constraints
Source: Entropy (Basel). 2018 Mar 30;20(4):240. doi: 10.3390/e20040240 (PMC7512755; doi:10.3390/e20040240)
Supplement: Supplementary file 1 [file entropy-20-00240-s001.zip › IdepFuns/READMEki2018.pdf]

R code for I_{dep} and I_{mmi} PIDs for Gaussian systems

These notes describe how to use the package R to compute PIDs for Gaussian systems[1]. The output for the numerical examples in the paper was produced using version 3.4.3 of R, which can be downloaded from `http://cran.r-project.org`.

A script for running Examples 5 and 6 is available in the folder IdepFuns, in the file, IdepGscript.txt. Once R is loaded, the user is required to set a working directory and then to load the functions that are contained in the file, IdepGaussR.txt.

Commands given in IdepGscript.txt can then be run by copying and pasting them into R and running them. Data used in examples 5 & 6 in the paper are provided in this file and .

The folder IdepFuns contains the definitions of four functions, given as follows

Function	Inputs	Outputs
idepGU	p, q, r	I_{dep} and I_{mmi} PIDs (univariate)
idepGM	sizes, mat	I_{dep} and I_{mmi} PIDs (multivariate)
DevTestU	p, q, r, n	Model names and p-values
DevTestM	sizes, mat, n	Model names and p-values

where

- p is the correlation between X_0 and X_1
- q is the correlation between X_0 and Y
- r is the correlation between X_1 and Y
- n is the number of observations (sample size)
- sizes is a numeric list of the values n_0, n_1, n_2
- mat is a positive definite covariance or correlation matrix

[1] Kay & Ince (2018): Exact partial information decompositions for Gaussian systems based on dependency constraints (*submitted*)
